# Magnetic domain wall gratings for magnetization reversal tuning and confined dynamic mode localization

**DOI:** 10.1038/srep30761

**Published:** 2016-08-04

**Authors:** Julia Trützschler, Kadir Sentosun, Babak Mozooni, Roland Mattheis, Jeffrey McCord

**Affiliations:** 1 Institute for Materials Science, Kiel University, Kiel, Germany; 2 Leibniz Institute of Photonic Technology IPHT Jena, Jena, Germany

## Abstract

High density magnetic domain wall gratings are imprinted in ferromagnetic-antiferromagnetic thin films by local ion irradiation by which alternating head-to-tail-to-head-to-tail and head-to-head-to-tail-to-tail spatially overlapping domain wall networks are formed. Unique magnetic domain processes result from the interaction of anchored domain walls. Non-linear magnetization response is introduced by the laterally distributed magnetic anisotropy phases. The locally varying magnetic charge distribution gives rise to localized and guided magnetization spin-wave modes directly constrained by the narrow domain wall cores. The exchange coupled multiphase material structure leads to unprecedented static and locally modified dynamic magnetic material properties.

Magnetic domains are generated in order to reduce the overall free energy, mainly the magneto-static energy, in magnetic systems^1^,^2^. Thus, without the application of external magnetic fields, magnetic domains and domain walls are unavoidable in patterned soft-magnetic material systems. In soft-magnetic thin films, typically Néel-type domain walls are formed because of the minimization of demagnetizing energy within the domain walls^1^,^3^. Néel walls consist of a narrow core and a wide tail region^2^. Over a wide thickness range, from around 20 nm to 100 nm, cross-tie walls consisting of a system of 90° Néel domain walls are energetically favoured and formed instead of 180° domain walls. The understanding of domain wall behavior is important, as measurable magnetic properties, such as the coercive field in soft-magnetic films, are directly related to the internal domain wall structure and the domain wall density. The pure existence of domain walls in soft-magnetic materials also provokes changes in dynamic properties and permeability spectra^4^,^5^. The impact of the inevitable magnetic domain walls on the dynamic properties of patterned soft-magnetic thin films has attracted attention due to increasing data rates in magnetic recording^6^,^7^ as well as for applications like high frequency inductors^8^. Domain walls are also assumed to play an important role for the excitations of spin waves, even in large structured magnetic elements^9^,^10^. Moreover, the use of single magnetic domain walls in thin films for magnonic applications^11^ has gained recent attention^12^,^13^. The number and the density of magnetic domain walls, however, are hard to manipulate and vary with the history of magnetic field application. For an equilibrium magnetic domain state, the domain spacing is determined by balancing magnetic anisotropy, magnetic exchange, and magnetic stray field energy contributions.

To intentionally tailor the static and dynamic magnetization behavior of extended unpatterned magnetic thin films by domain walls, domain walls need to be positioned in a controlled way within a magnetic thin film structure. Artificial domain wall structures can be achieved by strain mediation in multiferroic ferroelectric-ferromagnetic heterostructures incorporating ferromagnetic magnetostrictive layers in a bottom-up approach^14^,^15^. In this context it was found that 90° domain walls with various configurations can be formed, where the control of the domain wall spacing and the strength of magneto-elastically induced anisotropy are determined by the coupling to the domain structure of the ferroelectric substrate. Generation of artificial magnetic domain patterns and domain walls in thin films can also be accomplished by local ion irradiation^16^ in a top-down approach. Previous studies have demonstrated the setting of exchange bias directions in thin films^17^,^18^ and patterning of extended thin films into stripe-like magnetic microstructures^19^,^20^ with antiparallel unidirectional anisotropy directions. The latter structures are unique because a magnetic domain and a domain wall pattern can be imprinted directly into the magnetic material and, thus, a reproducible nucleation and positioning of magnetic domain walls in a high density arrangement can be obtained. A multi-domain state is formed without the need for magnetostatic interaction, but through spatially varying anisotropy. Local writing of individual unidirectional anisotropy patterns has been also achieved by scanning probe lithography^21^.

Here, configurational effects driven by magnetic charges at imprinted 90° and 270° artificial domain walls of different types are used to alter the magnetization response of extended sample geometries in a non-linear way. An initial unidirectional anisotropy (Fig. 1(a)) in ferromagnetic/antiferromagnetic Ni_81_Fe_19_/Ir_23_Mn_77_ thin films is set during film deposition in the presence of a magnetic in-plane field and an orthogonal alignment of exchange bias is obtained by subsequent light He-ion irradiation. A magnetization modulated landscape from domain walls is attained by varying the exchange bias in thin films in a stripe-like fashion with different orientation (Fig. 1(b,c)). Due to the imprinted unidirectional anisotropy network, magnetic structures are generated with magnetization vectors of adjacent domains pointing along and towards each other, respectively, in alternating head-to-tail-to-head-to-tail (〈+ − + −〉, Fig. 1(b)) and head-to-head-to-tail-to-tail (〈+ + − −〉, Fig. 1(c)) unidirectional anisotropy configurations. For the latter case, highly magnetically charged domain walls^22^ are formed. By the inter-stripe magnetic domain wall interaction not only quasi-static characteristics of the thin film, like effective magnetic anisotropy as well as magnetization and magnetoresistance response, are uniquely altered, but also dynamic properties such as confined precessional frequency modes in the extended films are introduced. The modifications of magnetic behavior are explained by coupling via variable magnetic charges from the domain walls, an effect that is adjusted by altering the micromagnetic interface density in the periodically modulated magnetization patterns. By varying the domain wall densities, the local dynamic domain wall properties can be probed directly by integral measurement techniques. The exact periodicity of the generated domain wall network allows for micromagnetic modeling of large sample size behavior. A complete picture of spatial and temporal evolution of magnetization within the films is derived.

## Results

### Magnetization process

The relevant quasi-static magnetization curves and corresponding anisotropic magnetoresistance (AMR) responses from non-irradiated and irradiated full films with the exchange bias direction oriented at ±*π*/4 to the applied magnetic field are shown in Fig. 2. The exchange bias fields for the as-deposited and the fully irradiated samples are *H*_eb,dep_ = 2.04 kA/m and *H*_eb,irr_ = 1.7 kA/m, respectively. The maximum AMR ratio AMR_max_ for the as-deposited sample is AMR_max,dep_ = 0.023. AMR_max,irr_ = 0.022 for the irradiated sample is of equivalent value. No noticeable difference in saturation magnetization was detected. In accordance with the imprinted direction of exchange bias, the net magnetization in remanence is *M*_*r*_/*M*_s,±*π*/4_ ≈ 0.7 (Fig. 2(a–d)) for the non-irradiated as for the irradiated sample. The amplitude of the AMR response at the coercive fields indicates a perpendicular alignment of magnetization relative to the applied magnetic field direction during switching. A relative remanent AMR response AMR_r_/AMR_max_ of AMR_r,±*π*/4_/AMR_max_ ≈ 0.5 is obtained. The AMR amplitude peaks at the coercive field *H*_c_ with AMR/AMR_max_ close to unity. The findings indicate reversal by coherent magnetization rotation. The coherent rotation of magnetization is affirmed by high resolution magneto-optical Kerr microscopy domain imaging^23^, where no magnetic domain activity was observed. Analyzing the magnetization response together with the AMR response provides a complete picture of angular magnetization behavior. An ion irradiation induced rearrangement of the equilibrium state of magnetization from −*π*/4 to −3*π*/4 with similar magnetic film properties is achieved.

**Figure 1:**
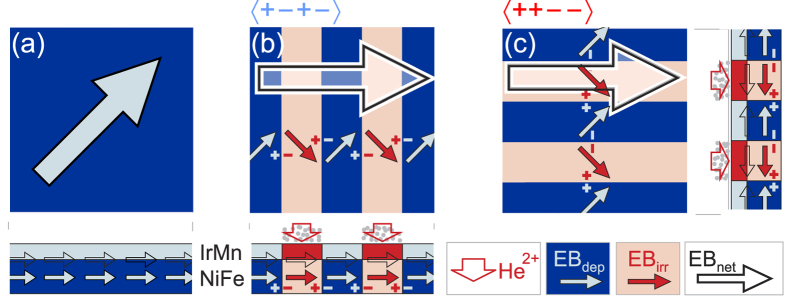
Ir_23_Mn_77_(7 nm)/Ni_81_Fe_19_(50 nm) sample structure and lateral state of exchange bias for (**a**) an as-deposited (*dep*) homogeneous state of exchange bias, (**b**) a head-to-tail-to-head-to-tail (〈+ − + −〉) and (**c**) the alternating head-to-head-to-tail-to-tail (〈+ + − −〉) unidirectional anisotropy configuration after local He-ion irradiation (*irr*) in an applied magnetic field aligned perpendicular to the as-deposited direction of exchange bias. The directions of exchange bias for the as-deposited EB_dep_, the irradiated regions EB_irr_, and the net directions of resulting exchange bias EB_net_ are indicated.

**Figure 2:**
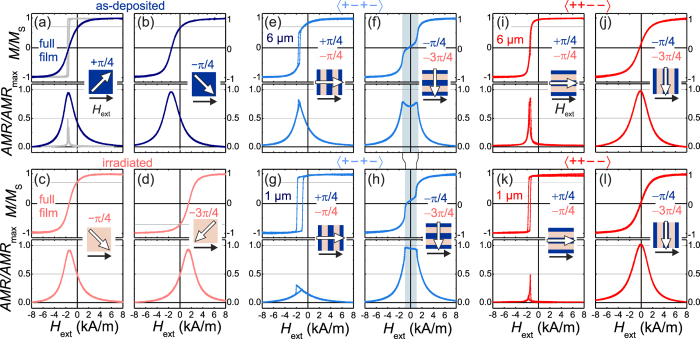
Magnetization curves *M(H*)/*M*_*s*_ = *cos* (*θ*) and corresponding magnetoresistance (AMR) AMR(*H*)/AMR_max_ = sin^2^(*θ*) response of an as-prepared full film with the magnetic field *H*_ext_ aligned under an angle of (**a**) *θ*_*dep*_ = *π*/4, and (**b**) *θ*_*dep*_ = −*π*/4 with respect to the initial direction of exchange bias. Corresponding data for an ion irradiated full film with (**c**) *θ*_*irr*_ = −*π*/4 and (**d**) *θ*_*irr*_ = −3*π*/4 relative to the rearranged unidirectional anisotropy (*π*/4 and −*π*/4 to the original direction of exchange bias). The data for applying a field along the direction of exchange bias is indicated in (**a**). Corresponding *M(H*) and AMR curves for structures with a stripe width of (**e,f**,**i,j**) 6 *μ*m and (**g,h,k,l**) 1 *μ*m, and with (**e–h**) head-to-tail-to-head-to-tail (〈+ − + −〉) and (**i–l**) head-to-head-to-tail-to-tail (〈+ + − −〉) unidirectional anisotropy configurations with the magnetic field *H*_ext_ aligned perpendicular [(**e,g,j,l**)] and parallel [(**f,h,i,k**)] to the stripe axis. The magnetic field direction, stripe orientation, and direction of net exchange bias EB_net_ are sketched. The angles of EB_dep_ and EB_irr_ relative to the direction of *H*_ext_ are indicated (see also Fig. 1).

Exemplary magnetization and AMR response curves of the patterned thin film with the head-to-tail-to-head-to-tail (〈+ − + −〉, Fig. 1(b)) and head-to-head-to-tail-to-tail (〈+ + − −〉, Fig. 1(c)) alignments of exchange bias for an external magnetic field *H*_ext_ perpendicular and parallel to the long axis of the stripes are shown in Fig. 2(e–h,i–l), respectively. For the 〈+ − + −〉 structures and applying the external magnetic field perpendicular to the stripe orientation, the bi-modal magnetic structure with the largest stripe width of 6 *μ*m switches at *H*_*c*_ ≈ 1.5 kA/m (Fig. 2(e)). The remanent magnetization increases as compared to the single phase material, thus the net magnetization pointing along the median direction of magnetization increases. This indicates an increased tilting of magnetization in the direction perpendicular to the stripe axis. The magnetization loop of the 1 *μ*m stripe width displays similar behavior (Fig. 2(g)). Yet the remanent magnetization increases further, pointing to a further increase of alignment of magnetization perpendicular to the grating’s long axis and the magnetic phase borders. Applying the field parallel to the grating results in a pronounced two-staged magnetization curve for both stripe widths. The two-step magnetic hysteresis loop displays an intermediate magnetization plateau at low external fields (Fig. 2(f)) and the plateau width decreases with decreasing stripe width (Fig. 2(h)) (see also Supplementary Fig. S1). Probing the transversal magnetization component by AMR clarifies the overall magnetization behavior. Setting the external magnetic field perpendicular to the stripes’ long axis leads to an AMR response with a well-defined maximum at the switching field. The decrease of AMR_r_/AMR_max,π/4_ for 6 *μ*m and 1 *μ*m stripe widths as compared to the AMR_r_/AMR_max,π/4_ values shown in Fig. 2(a–d) confirms the transversal alignment of magnetization relative to the orientation of the grating. For narrower stripes, the remanent and switching ratio AMR_r_/AMR_max,π/4_ further decreases. The magnetization straightens perpendicular to the stripes’ long axis. Analyzing the AMR response shows that the net magnetization is tilted away from the originally imprinted ±45° to on average ±38° for a stripe width of 6 *μ*m and down to ±24° for the 1 *μ*m grating. Thus, for the 〈+ − + −〉 configuration the net magnetization and effective exchange bias increases perpendicular to the induced magnetic phase boundaries with smaller feature size.

Different modifications of magnetization reversal behavior are obtained for the highly magnetically charged 〈+ + − −〉 configurations as shown in Fig. 2(i–l). Application of the external magnetic field parallel to the stripes’ long axis leads to an almost instantaneous switching of magnetization for the large stripe width (6 *μ*m, Fig. 2(i)). Notably, a slightly increased effective exchange bias is obtained for 1 *μ*m stripes (Fig. 2(k)). The reversal process displays almost no change in AMR signal at the switching field in accordance with the easy axis loop obtained under a different field angle (+0*π* in Fig. 2(a)). For the 〈+ + − −〉 configuration, the net magnetization and effective exchange bias increase parallel to the induced magnetic phase boundaries with smaller feature size. With perpendicular field alignment, non-hysteretic hard axis loops without the development of plateaus around zero field are obtained for both stripe widths (Fig. 2(j,l)). The corresponding AMR signals show a clear maximum at zero field close to unity of AMR(*H*)/AMR_max_, implying an alignment of magnetization along the magnetic grating at zero magnetic field for the 〈+ + − −〉 configuration. Yet the AMR value is slightly higher and the magnetization is aligned nearly to the stripe axis for the 1 *μ*m stripes. From the AMR response, the tilting of magnetization away from the imprinted ±45° is estimated to be on average ±33° for a stripe width of 6 *μ*m and down to at least ±40° for the 1 *μ*m grating, but the opposite for the 〈+ − + −〉 case.

### Magnetization structure

**Figure 3:**
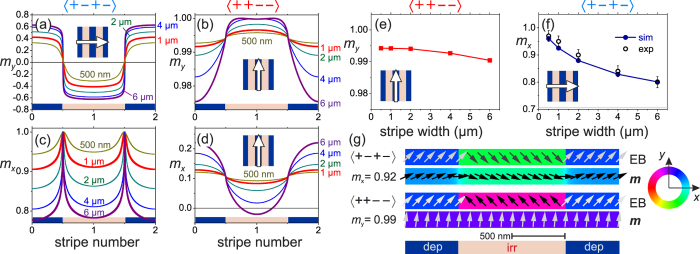
Calculated magnetization components (**a,b**) *m*_*y*_ and (**c,d**) *m*_*x*_ for the (**a,c**) 〈+ − + −〉 and the (**b,d**) 〈+ + − −〉 stripe configuration, varying across the stripes. The individual stripe widths are indicated. Corresponding average magnetization components *m*_*y*_ and *m*_*x*_ for the 〈+ + − −〉 and 〈+ − + −〉 configurations are given in (**e,f**), respectively. (**g**) Exemplary data of the calculated magnetization configuration across the magnetic phase boundaries for a stripe width of 1 *μ*m for the 〈+ − + −〉 and 〈+ + − −〉 configuration. The orientation of stripes and EB_net_ is sketched (*H*_ext_ = 0).

Micromagnetic simulations of the ground-state of the magnetization configurations merely based on the full film magnetic properties are used to quantify the states of magnetization. A summary of the results is displayed in Fig. 3. The profiles of magnetization components across the stripes for the 〈+ − + −〉 configurations show all the characteristics of overlapping Néel wall structures (Fig. 3(a)). With decreasing stripe width, the alteration of *m*_*y*_ across the domain wall and the stripe border reduces. Correspondingly, the *m*_*x*_-component of magnetization (Fig. 3(c)) increases. In agreement with the experimental results, the magnetization aligns more perpendicular to the magnetic phase boundaries with decreasing stripe width. For the 〈+ + − −〉 configurations, *m*_*y*_ is close to unity even for the greatest stripe width (Fig. 3(b)). This is a direct consequence of the charged 〈+ + − −〉 magnetization structure, leading to long tail Néel wall structures. The magnetization aligns along the stripe axis. The magnetization component *m*_*x*_ perpendicular to the stripe axis varies only slightly (Fig. 3(d)). The offset of *m*_*x*_ from zero is attributed to the differences in exchange bias amplitude of the non-irradiated and the irradiated phase. In accordance with the zero field data displayed in Fig. 2(i,k), *m*_*y*_ is close to unity for the 〈+ + − −〉 configuration (Fig. 3(e)). Comparing the experimentally (see Fig. 2(e,g)) obtained integral values of *m*_*x*_ with the results of the micromagnetic simulations for the 〈+ − + −〉 configuration from various stripe widths, nearly perfect agreement is obtained (Fig. 3(f)). This proves the accuracy and the validity of the micromagnetic simulation data in order to describe the magnetization behavior. The lateral directions of exchange bias together with the calculated alignment of magnetization for the 〈+ − + −〉 and 〈+ + − −〉 structures are shown in (Fig. 3(g)). The special micromagnetic structures also modify the magnetization processes in a unique way.

### Magnetic domain behavior

**Figure 4:**
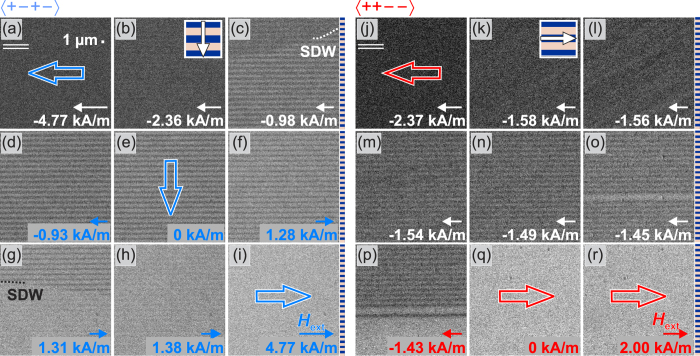
Magnetic domain behavior for the (**a–i**) 〈+ − + −〉 and the (**j–r**) 〈+ + − −〉 configuration with magnetic field orientation parallel to the stripe axis. The stripe width is 1 *μ*m. The external magnetic field axis (*H*_*ext*_) and the magneto-optical sensitivity axis (||) are indicated. Net directions of magnetization for the whole structure are exemplarily sketched as hollow arrows. The orientation of stripes and EB_net_ is sketched in (**b,k**). The direction and amplitudes of *H*_ext_ are indicated.

The variable magnetic reversal behavior of the magnetic domain wall loaded parts is demonstrated by high resolution magneto-optical Kerr effect microscopy in the longitudinal mode^23^. The reversal modes perpendicular and along the stripe axis for a stripe width of 1 *μ*m are shown in Fig. 4 (for a stripe width of 6 *μ*m see Supplementary Fig. S2).

With small stripe width and high density of imprinted domain walls, the interaction between the magnetic phases leads to fundamental changes in the magnetization reversal behavior as compared to a single phase magnetic thin film. For the 〈+ − + −〉 configuration with the magnetic field parallel to the grating (compare to Fig. 2(h)), the magnetization distribution unfolds by the movement of a superdomain wall (SDW) across the stripes below a threshold field (Fig. 4(c)). The magnetic charge stabilized structure is then stable over a wide magnetic field range (Fig. 4(c–g)). Generation and annihilation of the magnetically modulated structure takes place by magnetization rotation in the irradiated and the non-irradiated stripes. Nonetheless, limited low angle domain wall motion across the stripe borders occurs in the intermediate low field regime (Fig. 4(d–f)). The charge stabilized magnetization regime coincides with the low permeability central regime displayed in Fig. 2(h).

For the 〈+ + − −〉 configuration with the alignment of net magnetization along the grating, a very different reversal behavior is observed (Fig. 4(j–r)). With the magnetic field aligned along the grating of exchange bias (see Fig. 2(k)), the magnetization first rotates and then forms a modulated magnetization structure. The magnetization unfolds to the modulated structure close to the magnetic switching field (Fig. 4(m,n)). Yet reversal takes place by switching of the magnetization in individual stripes (Fig. 4(o,p)). The unfolding occurs through individual stripe reversal (Fig. 4(m–o)) in a non-collective way. No low angle domain wall motion across the stripes takes place in the intermediate low field regime.

### Domain wall dynamics

**Figure 5:**
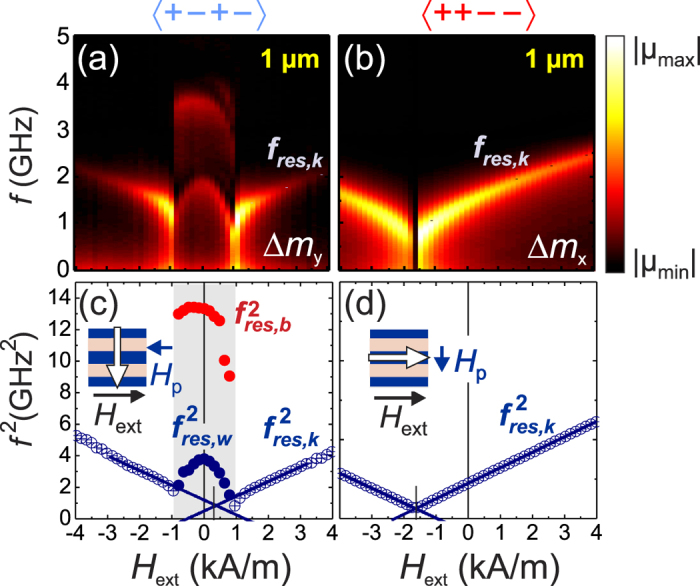
Dynamic permeability spectra maps *μ*(| *f* |, *H*_ext_) of films with (**a**) 〈+ − + −〉 and (**b**) 〈+ + − −〉 exchange bias modulation at a stripe width of 1 *μ*m. (**c,d**) Dominant precessional frequencies 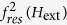 derived from the data in (**a,b**). The dynamic Kittel behavior is fitted. *H*_ext_ is applied parallel to the stripe axis. The direction of the pulsed magnetic field *H*_p_ is indicated. (The two other corresponding configurations are shown in Supplementary Fig. S3).

The dense magnetic domain configurations also alter the dynamics of magnetic properties significantly. For dense domain wall gratings the overall dynamics are dominated by the domain walls and not the matrix phase. The dynamic permeability spectra of the samples were obtained by pulsed inductive microwave magnetometry (Fig. 5). For the data shown the films are oriented with the dynamic pulse field perpendicular to the net magnetization, aligned parallel for 〈+ − + −〉 to the stripe long axis, respectively perpendicular for 〈+ + − −〉. For the 〈+ − + −〉 structure, two distinct regions occur (Fig. 5(a,c)). Above a negative and positive threshold field, the precessional frequency displays a regular Kittel mode with a collective single precessional frequency *f*_res,k_. The Kittel region reflects the single domain status of the sample (Figs 2(h) and 4(a–c,g–i)). Yet the central region, where the grated multi-domain wall state is in place (plateau region in Figs 2(h) and 4(c–g)), displays a unique and strongly modified dynamic magnetization behavior. Two distinct precessional frequency peaks *f*_res,w_ and *f*_res,b_ are seen in Fig. 5(a). Both precessional frequencies exhibit a maximum at zero magnetic field. The slight asymmetry of the precessional frequency dependence in the bi-modal region and the frequency minima reflect the asymmetric effective exchange bias fields of the individual stripe fractions. This also shows in the slight shift of extrapolated Kittel modes from both high field branches.

For the 〈+ + − −〉 structure with nearly homogeneous magnetization (Fig. 3), only a single Kittel mode is seen for the complete magnetic bias field range (Fig. 5(b,d)). In agreement with the effective net exchange bias along the stripe axis in the stripe 〈+ + − −〉 structures (Fig. 2(h)), the 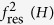 curve is shifted to negative field values.

**Figure 6:**
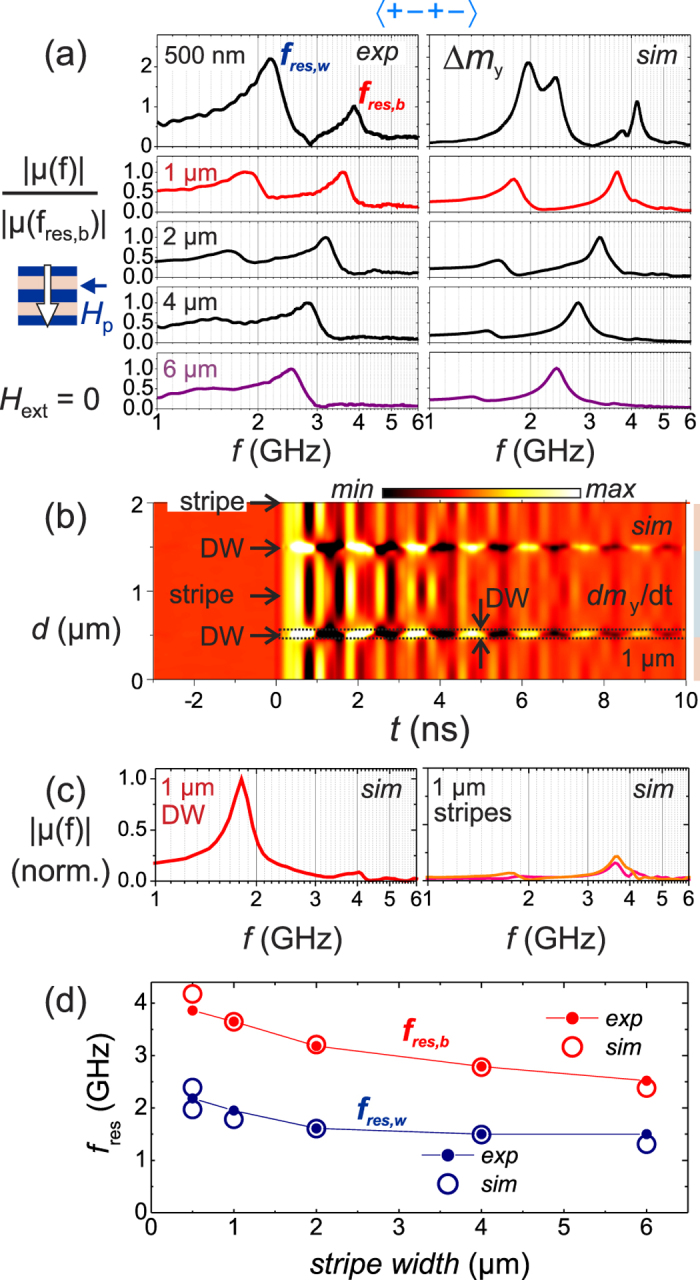
(**a**) Experimental and simulated permeability spectra |*μ(H*_bias_)|/|*μ(f*_res,b_)| of films with 〈+ − + −〉 configuration and with a stripe width of 500 nm up to 6 *μ*m. (For other configurations and a stripe width of 1 *μ*m, see Supplementary Fig. S4). (**b**) Simulated spatial development *dm*_y_/*dt* with time *t* for a stripe width of 1 *μ*m (*t* = 0 ns corresponds to the onset of magnetic field pulse). (**c**) Calculated local permeability spectra at the location of the domain wall (DW) and in the center of the stripes (as indicated in (**b**)). (**d**) Comparison of experimental and modeled frequency positions of precessional peaks at *f*_res,w_ and *f*_res,b_.

Clarity on the bi-modal dynamic behavior is obtained from direct comparison of dynamic inductive and modeling data. Agreement of simulation and experiments over the whole frequency range is demonstrated in Fig. 6. The data is normalized to the permeability amplitude of the higher frequency mode at *f*_res,b_. A bi-modal dynamic response is observed for all stripe widths (Fig. 6(a)). Both modes shift to higher frequencies for smaller stripe widths and increasing domain wall density and magnetic charge density. With the increase of the domain wall density, the amplitude of the lower frequency modes at *f*_res,w_ relative to *f*_res,b_ increases strongly, indicating a spatial localization of the low frequency mode at the imprinted domain walls of the grated film structure. This assumption is proven by analyzing the calculated spatial distribution of the dynamic magnetization modes for a stripe width of 1 *μ*m, as shown in Fig. 6(b) (for corresponding time domain data of *m(t*) see Supplementary Fig. S5). A spatial localization of the low frequency mode *f*_res,w_ in the core of the domain walls at the stripe borders becomes obvious. The mode localization results from the formation of a potential well for the dynamic modes in the inhomogeneous internal magnetic field of the domain wall, similar to the edge regions of nearly saturated magnetic stripes^24^. The overall amplitude of the high frequency mode *f*_res,b_, clearly located in the domain wall tail region in the center of the individual stripes, is significantly lower. An analysis similar to Fig. 6(a) but now for the domain wall core and center of the stripes is shown in Fig. 6(c). The overall excitation inside the domain walls is much higher than in the stripes. Only a weak crosstalk from the stripe into the domain wall region (and vice versa) is seen from the data. A channel with reduced dynamic activity bordering the domain walls can be identified in Fig. 6(b). Yet the total contribution of the stripe bulk mode *f*_res,b_ to the overall dynamic behavior Fig. 6(b) is still comparable to the domain wall mode contribution at *f*_res,w_ due to the limited volume of the constricted domain walls. Thus, in agreement with the experiments, the contributions from the domain walls increase and dominate for high domain wall densities. A conclusive comparison of the exhibited frequencies of the modes is displayed in Fig. 6(d), confirming the consistency of the experimental and modeling results.

## Discussion

Artificial Néel domain walls with different magnetostatic contributions can be generated by anisotropy patterning in exchange biased thin films. Longitudinal modulated head-to-tail-to-head-to-tail 〈+ − + −〉 and magnetically charged transversal head-to-head-to-tail-to-tail 〈+ + − −〉 domain structures are formed. The altered magnetic structure results in magnetic domain wall dominated magnetization behavior for high packing densities. The overlapping domain wall structures give rise to reorientation of the magnetization away from the imprinted magnetic anisotropy direction and to formation of magnetic superdomains. The nucleation and annihilation of the dense domain wall structures result in highly non-linear magnetization processes. By varying the domain wall density, the domain wall properties can be probed by integral magnetic measurement techniques. Magnetization reversal and magnetoresistive response are tailored with the inclusion of domain wall networks.

Local dynamic magnetic modes with increased amplitude relative to the surrounding material matrix are pinned at the core of the magnetic domain walls. The dynamic activity is located at the borders of the magnetic phases. The width of the dynamically active area is thus defined by the domain wall core width, but not by the resolution of the patterning process. A dynamic path in the nanometer range is thus introduced by a hybrid top-down in combination with a bottom-up physical mechanism, by structuring and ion radiation and from the domain wall width that is defined by the magnetic material properties. The core Néel domain wall width *δ*_*c*,*w*_ for soft magnetic materials is typically below the magnetic film thickness, corresponding to *δ*_*c*,*w*_ ≤ 50 nm in our case. Yet due to magneto-static energy contributions long-range magnetic tail structures with a tail width *δ*_*t*,*w*_ extending over several micrometers exist. Estimating the effective domain wall core width *δ*_*FWHM*,*w*_ from the full width at half maximum (FWHM) of the obtained Néel wall profiles from micromagnetic simulations leads to an effective width of between *δ*_*FWHM*,*w*_ ≈ 105 nm and *δ*_*FWHM*,*w*_ ≈ 150 nm for a stripe width of 500 nm and 6 *μ*m (Fig. 3(c)), respectively. *δ*_*FWHM*,*w*_ defines the confinement of the domain wall mode (marked in Fig. 6(b)).

To summarize, domain wall networks of high density are generated in soft magnetic thin films by local light ion irradiation by which head-to-tail-to-head-to-tail and head-to-head-to-tail-to-tail domain structures with high packing densities are formed. The annihilation of domain walls due to the increase in total energy with increasing domain wall density is prohibited by the induced anisotropy pattern. The high integration of domain walls in homogeneous thin films of different angles shown here is only achievable through lateral shaping of the magnetic energy landscape. Writing high density domain walls of different orientations allows for lateral anisotropy modifications and for domain wall guided magneto-dynamics. The shown periodically patterned structures might also be applicable for magnonic grating couplers for the directional emittance of short wavelength spin-waves^25^,^26^ as well as for domain wall based spin wave sources^27^. A variation of the scheme presented could include more complicated lateral arrangements of domain wall networks. Forming a network of different types of domain wall structures achievable by multilayering of magnetic layers is one further option to obtain unique magnetic static and dynamic magnetic properties. Possible applications vary from magnetic sensors with tailored hysteresis and field sensitivity functions to high frequency inductive applications to domain wall guided magnonic applications.

## Methods

### Thin film samples

Extended Ta(5 nm)/Ni_81_Fe_19_(50 nm)/Ir_23_Mn_77_(7 nm)/TaN(5 nm) thin films were deposited by DC magnetron sputtering under UHV conditions with a base pressure below 2 × 10^−8^ Torr by using 6N Ar at a pressure of 10^−3^ Torr on oxidized Silicon substrates. An initial unidirectional anisotropy in the layer stack was set during the sputtering process by a homogeneous magnetic field of 4 kA/m. No post annealing steps were performed. The Ta buffer layer acts as a seed layer for the Ni_81_Fe_19_ to guarantee a well-defined 〈111〉 texture. The top TaN layer prevents corrosion of the thin film stack. The sample size is (1 cm × 1 cm), allowing for complementary integral as well as laterally resolved local analysis.

### Local ion irradiation

Through photolithographic masking, photoresist stripes aligned either at +*π*/4 or −*π*/4 to the initial exchange bias direction was introduced. The stripe width was varied from 6 *μ*m down to 500 nm, being well below the distance of the extended magnetic Néel domain wall tails^2^. After this, full field Helium ion bombardment with an energy of 10 keV and a fluence of 1.2 ⋅ 10^15^ ions/cm^2^ was performed by which a reorientation of exchange bias in accordance with the state of magnetization in the non-masked regions was achieved. To alter the initial unidirectional anisotropy in the ion irradiated areas, an external in-plane magnetic saturation field (*H*_sat_ = 26 kA/m) was applied during irradiation. The field was oriented *π*/2 to the initial exchange bias direction. By doing this, artificial magnetic domain patterns were introduced, resulting in stripe-like two-dimensional structures with alternating directions of exchange bias^28^. For vertically aligned stripes, the local tailoring of exchange bias led to a creation of a head-to-tail-to-head-to-tail configuration of anisotropy and magnetization (〈+ − + −〉, Fig. 1(b)). For the horizontal stripe setting, an alternating head-to-head-to-tail-to-tail magnetization configuration was imprinted (〈+ + − −〉, Fig. 1(c)).

### Static and dynamic characterization

Quasi-static magnetization curves were obtained by inductive magnetometry carried out 1 cm × 1 cm at a frequency of 10 Hz. From this the component of magnetization *m*_||_


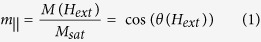


parallel to the applied magnetic field was obtained. Supplementary measurements of the anisotropic magneto-resistance (AMR) were conducted at a frequency of 1 Hz by a four point van der Pauw measurement method^29^,


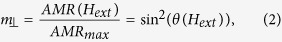


by which information about the transverse magnetization component *m*_⊥_ relative to the applied magnetic field was derived. The AMR amplitude was calibrated against the AMR response of stripe structures measured in a linear four probe setup on structured elongated stripe structures. The magnetic field resolution Δ*H* for the magnetometry and the AMR measurement is better than Δ*H* = 30 A/m. Laterally resolved information of the magnetization reversal was studied by high resolution magneto-optical Kerr effect microscopy in the longitudinal mode^23^.

The dynamic magnetic characteristics, e.g. magnetic permeability spectra, were obtained by pulsed inductive microwave magnetometry (PIMM)^30^ with an in-plane magnetic field pulse of *H*_*pulse*_ ≈ 1.5 A/m and a rise time of *t*_*rt*,10−90_ ≤ 50 ps. The measurements were performed with varying bias field *H*_*ext*_. The time domain data was transferred into the frequency domain by fast fourier transformation, from which the dynamic magnetic permeability spectra were obtained.

### Micromagnetic simulations

In order to resolve the different behavior of the 〈+ − + −〉 and the 〈+ + − −〉 domain wall phases, the dynamics of the two configurations were calculated by micromagnetic simulations^31^ based on the finite difference method, solving the Landau-Lifshits equation for the motion of the normalized magnetization *m*_*i*_ of the *i*^*th*^ discretization cell in the form


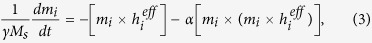


with *γ* being the gyromagnetic ratio and *α* the magnetic damping parameter of the material. No thermal effects were taken into account for the simulations. The in-plane cell size for the calculations ranged from 1.25 nm for the narrow stripe width of 500 nm to 15 nm for the wide stripe width of 6 *μ*m. The vertical dimension of the cell was the film thickness. A saturation magnetization of *μ*_0_*M*_*s*_ = 1 T and a magnetic damping parameter *α* = 0.01^32^ was used for the Ni_81_Fe_19_ layer in the calculations. The exchange stiffness constant was set to *A* = 1.05 × 10^−11^ J/m^33^. The laterally varying exchange bias parameters were derived from magnetic loop analysis of full film structures. No adjustments to the obtained experimental full film parameters were performed. The extended film (1 cm × 1 cm) magnetization dynamics were simulated by using periodic boundary conditions in the film plane with the pulse-field conditions as in the PIMM experiments.

## Additional Information

**How to cite this article**: Trützschler, J. *et al*. Magnetic domain wall gratings for magnetization reversal tuning and confined dynamic mode localization. *Sci. Rep.* **6**, 30761; doi: 10.1038/srep30761 (2016).

## Supplementary Material

Supplementary Information
