# Supplementary material for: Magnetic domain wall gratings for magnetization reversal tuning and confined dynamic mode localization
Source: Sci Rep. 2016 Aug 4;6:30761. doi: 10.1038/srep30761 (PMC4973262; doi:10.1038/srep30761)
Supplement: Supplementary Information [file srep30761-s1.pdf]

Magnetic domain wall gratings for magnetization reversal tuning and confined dynamic mode localization

Julia Trützschler,[1] Kadir Sentosun,[1] Babak Mozooni,[1] Roland Mattheis,[2] and Jeffrey McCord[1]

[1]Institute for Materials Science, Kiel University, Kiel, Germany
[2]Leibniz Institute of Photonic Technology IPHT Jena, Jena, Germany

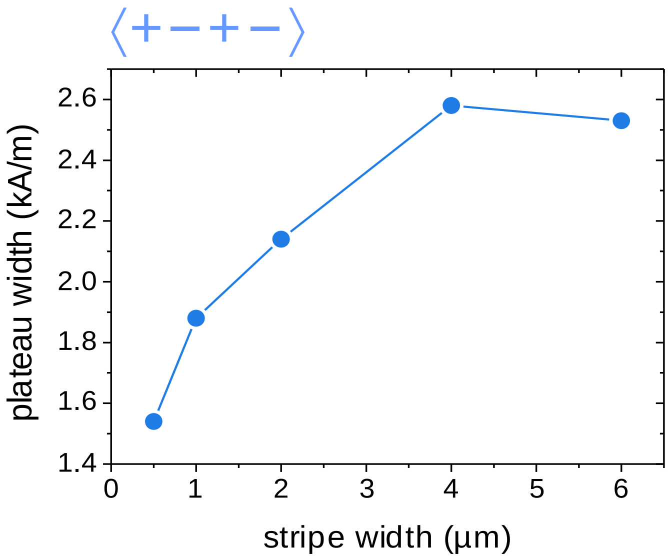

Fig. S1 Magnetic field plateau width from two-step magnetic hysteresis loop versus stripe width.

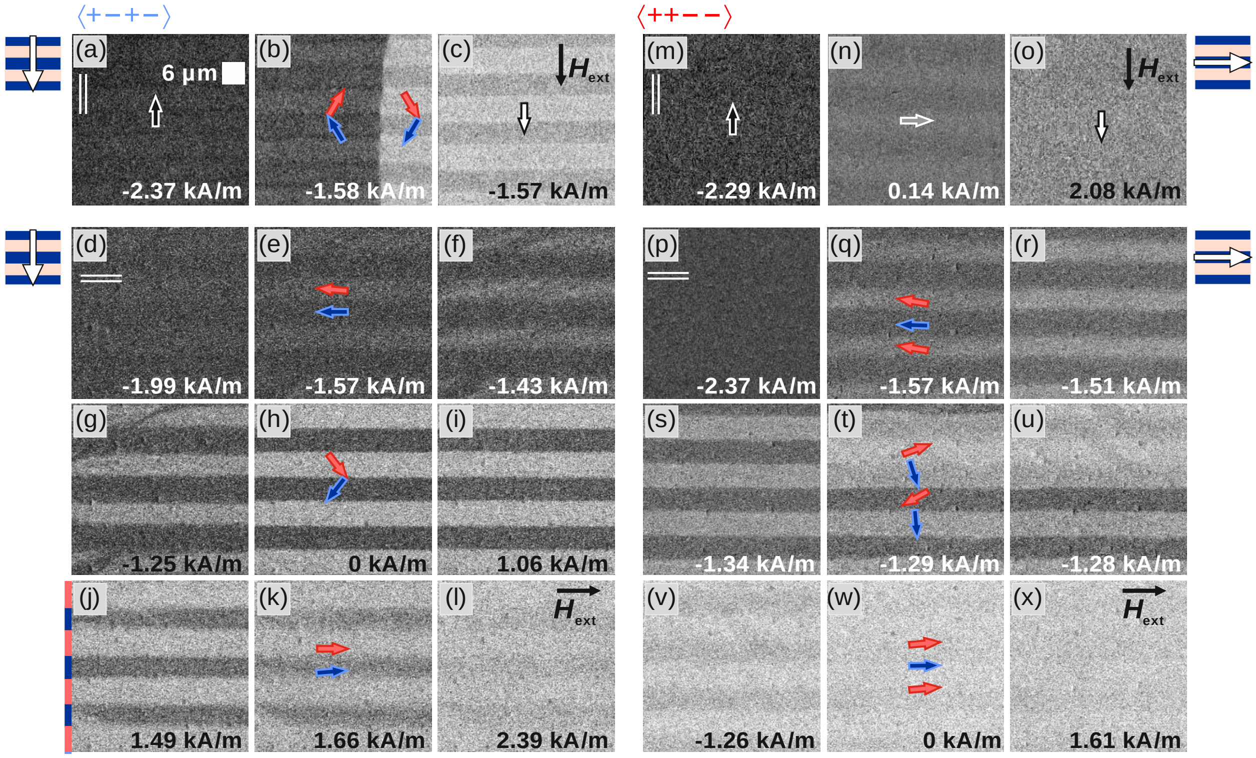

Fig. S2 Magnetic domain behavior for the (a)-(l) <+−+−> and the (m)-(x) <++−−> configuration with parallel and vertical magnetic field orientation. The stripe width is 6 µm. The external magnetic field H_{ext} axis as well as the magneto-optical sensitivity axis (||) are indicated. Net directions of magnetization for the whole structure and for the individual as-deposited and irradiated stripes are exemplarily sketched.

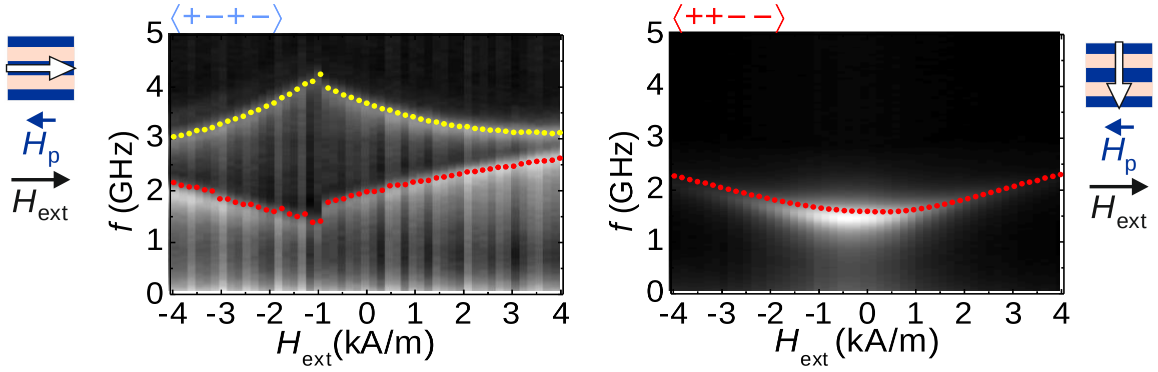

Fig. S3 Dynamic permeability spectra maps of films with (left) <+−+−> and (right) < ++−−> exchange bias modulation at a stripe width of 1 µm with the magnetic bias field applied perpendicular to the stripe axis. Stripe alignment, external magnetic field direction, and pulse field orientation are indicated.

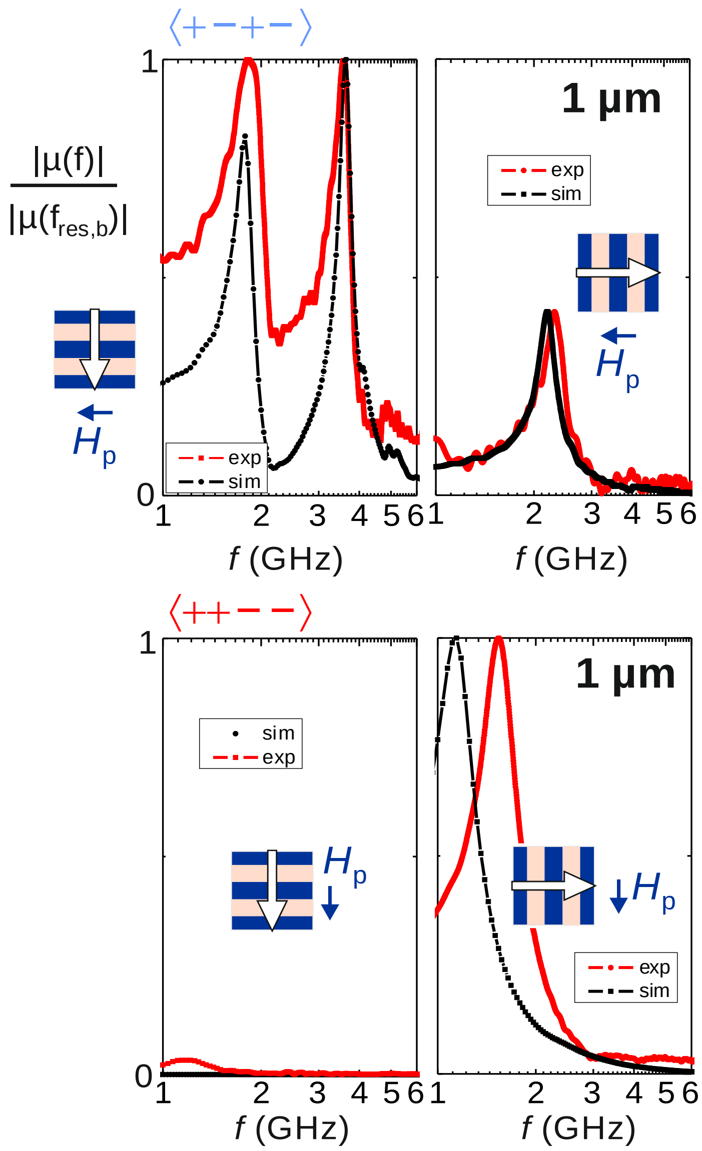

Fig. S4 Comparison of experimental and simulated permeability spectra $|\mu(f)|/|(\mu(f_{res,b})|$ of films with <+−+−> and <++−−> configuration and with a stripe width of 1 μm. The pulse field H_p is applied along and perpendicular to the stripe axis. Stripe alignment and pulse field orientation are indicated.

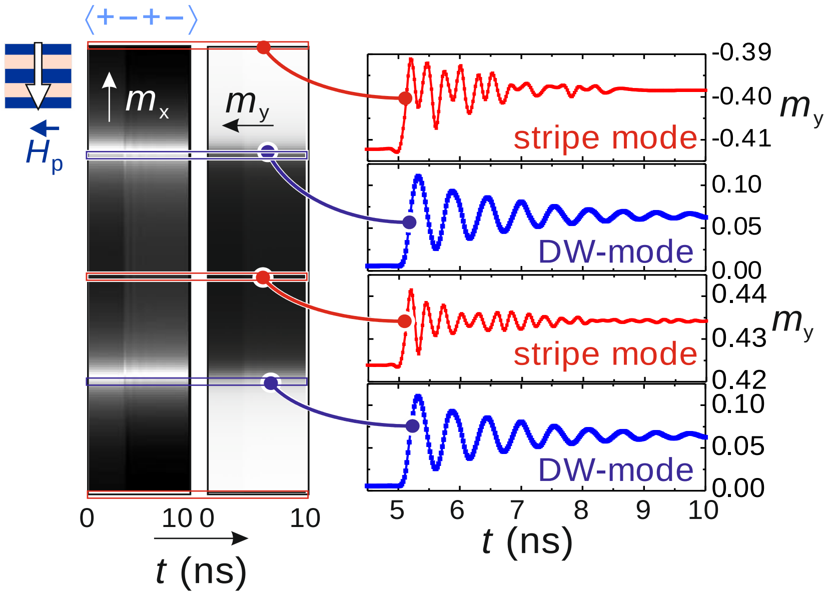

Fig. S5 Simulated spatial development of magnetization with time t for a stripe width of 1 µm (regions as indicated).
